# Huge pyogenic cervical cyst with endometriosis, developing 13 years after myomectomy at the lower uterine segment: a case report

**DOI:** 10.1186/1472-6874-14-104

**Published:** 2014-09-03

**Authors:** Katsutoshi Oda, Yuji Ikeda, Daichi Maeda, Takahide Arimoto, Kei Kawana, Masashi Fukayama, Yutaka Osuga, Tomoyuki Fujii

**Affiliations:** 1 Department of Obstetrics and Gynecology, The University of Tokyo, 7-3-1 Hongo, Bunkyo-ku, Tokyo 113-8655, Japan; 2 Department of Obstetrics and Gynecology, Kawakita General Hospital, 1-7-3 Asagayakita, Suginami, Tokyo 166-0001, Japan; 3 Department of Pathology, Kawakita General Hospital, 1-7-3 Asagayakita, Suginami, Tokyo 166-0001, Japan; 4 Department of Pathology, Akita University Hospital, 44-2 Azahasunuma Hiroomote, Akita City 010-8502, Japan; 5 Department of Pathology, The University of Tokyo, 7-3-1 Hongo, Bunkyo-ku, Tokyo 113-8655, Japan

**Keywords:** Cervical pyogenic cyst, Uterine fibroid, Myomectomy scar, Endometriosis

## Abstract

**Background:**

Surgical site infections are potential complications following open myomectomy. These infections usually develop immediately after the surgery, and are most often located in the myometrium. Pyogenic cervical cysts are rare and have not been previously reported to occur at the site of myomectomy.

**Case presentation:**

A 41-year-old nulligravida Japanese woman was referred to our hospital with a large cervical cyst (>15 cm in diameter). She had undergone a myomectomy 13 years previously, and the surgical site had extended to the endocervical gland. Standard blood tests did not show any evidence of inflammation. The patient underwent a total abdominal hysterectomy, which revealed that the cyst contained multiple components, including *Escherichia coli*, old blood, and evidence of endometriosis. A pathological review did not show malignant cells within the cyst. The pyogenic cyst originated from the upper anterior cervix, which was one of the sites involved in the previous myomectomy.

**Conclusion:**

We reported a huge pyogenic cervical cyst exhibiting signs of endometriosis, in the vicinity of the uterine scar from the open myomectomy. The previous surgery and endometriosis might have contributed to the formation of this rare pyogenic cyst.

## Background

Uterine fibroids are commonly observed, affecting >30% of women of reproductive age [1]. Fertility-sparing surgery (myomectomy) is one of the major treatment options for younger patients [2], although potential risks are associated with this treatment. Myomectomy is associated with a risk of recurrent uterine fibroids, pelvic adhesions, as well as complications at the incision site. Furthermore, myomectomy increases the risk of uterine rupture during labor, and commonly requires planned cesarean sections [3]. Unfortunately, post-cesarean section abscess formation has been reported at the site of uterine incision [4]. Moreover, intra-uterine infection may develop before or after cesarean section, and the risk of surgical site infection has been reported to increase after cesarean section [5]. However, no studies have reported the development of pyogenic cervical cysts, without signs of infection, after myomectomy. In the present report, we describe a rare case of a pyogenic cervical cyst, further complicated by endometriosis, arising 13 years after open myomectomy.

## Case presentation

**Figure 1:**
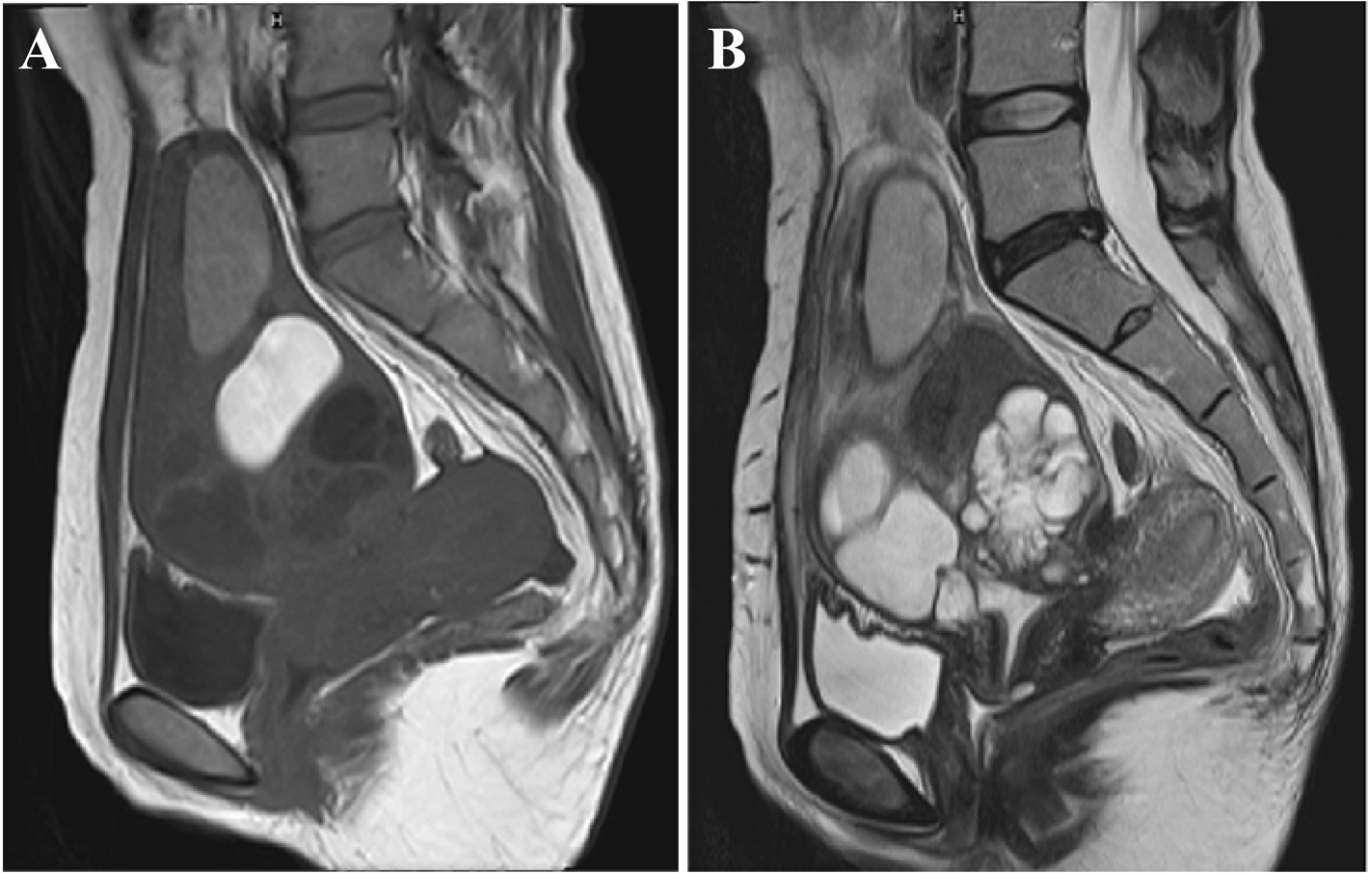
**Magnetic resonance imaging (MRI) of the pyogenic cervical cyst. (A)** a sagittal T1-weighted and **(B)** a sagittal T2-weighted imaging revealed a large cystic mass at the anterior cervix. The mass was a complex multilocular cyst, consisting of at least two components. The cranial part of the cyst contained two high-intensity cystic masses on the T1-weighted image, and the caudal part contained two multilocular masses that demonstrated low-intensity on the T1-weighted image and high-intensity on the T2-weighted image.

**Figure 2:**
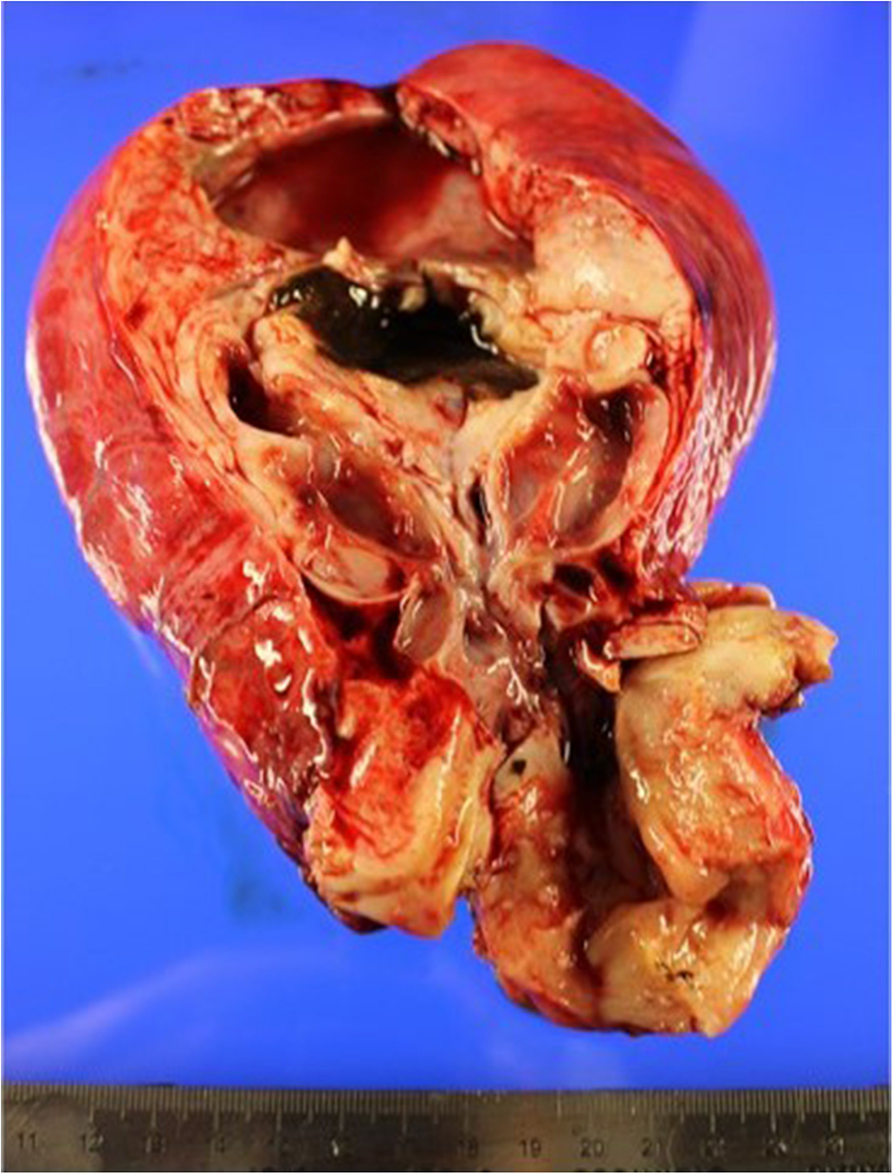
**Macroscopic findings of the cut surface of the pyogenic cervical cyst.** The distal part of the mass contained old blood, similar to that in an endometrial cyst, and the proximal part contained mucinous fluid, similar to a nabothian cyst or lobular endocervical glandular hyperplasia.

A 41-year-old nulligravida Japanese woman was referred to our hospital (Kawakita General Hospital, Tokyo, Japan) due to the presence of a large cervical mass. Thirteen years previously, she had undergone myomectomy for the treatment of multiple uterine fibroids in the lower uterine segment, including one weighing 1.4 kg. Penetration beyond the endocervical gland had occurred during resection, and the anterior endocervical canal had been opened during the surgery. Her original follow-ups were approximately once a year at another hospital, where cancer screening was performed using transvaginal ultrasonography and cytology of the cervix and endometrium. Eight years after the original surgery, she had undergone pelvic magnetic resonance imaging (MRI), which did not detect any abnormal masses in the uterine body or cervix. Twelve years after the original surgery, she underwent transvaginal ultrasonography, and no abnormal masses were detected.However, a pelvic examination, performed 13 years after her myomectomy, indicated marked enlargement of the cervix, which was suspected to be a cervical malignancy. The patient had experienced no symptoms prior to the examination, and given the lack of observed progression, we were unable to estimate the period over which the mass developed. Transvaginal ultrasonography indicated that the mass contained multilocular, hypoechoic lesions, with heterogeneous internal echogenicity. Furthermore, MRI confirmed the presence of a multilocular, irregularly shaped cystic mass (overall diameter, >15 cm) in the upper anterior cervix (Figure 1A and B). Computed tomography (CT) did not show any evidence of enlarged lymph nodes, ascites, or a distant tumor. Blood tests did not show elevated levels of inflammation or tumor markers. The patient’s white blood cell count was 6,000/mL and C-reactive protein level was <0.3 mg/dL. Levels of CA125, CA19-9, and squamous cell carcinoma antigen (SCC) were 32.4 U/mL, 6.4 U/mL, and 1.4 U/mL, respectively. Results from cytological examination of both the cervix and endometrium were negative for cancer.Therefore, we suspected that the mass was either a degenerated uterine fibroid, lobular endocervical glandular hyperplasia, or cervical malignancy. We performed a total abdominal hysterectomy, and dense adhesions were observed in the pelvis, particularly around the cervical mass. Moreover, the cervix was markedly enlarged, given the mass in the anterior wall. However, no additional abscesses or ascites were detected in the abdomen. A macroscopic examination revealed that the cystic mass originated from the upper anterior cervix, which was one of the sites involved in the original myomectomy. The mass consisted of 2 components, one containing an abscess within the soft solid tissue, and another that was filled with old blood (similar to an endometrial cyst) (Figure 2).

Following culture of the abscess tissue, *Escherichia coli* was identified as the causative bacteria. Microscopic examination revealed the coexistence of endometriosis, and an abscess was confirmed within the cyst, although malignant cells were not identified. An additional pathological review was performed at the University of Tokyo, which confirmed the absence of atypical or malignant cells. Thus, we diagnosed the mass as a pyogenic cyst, with endometriosis, which most likely occurred at the site of the uterine scar.

## Discussion

Surgical site infections are common complications of myomectomy, and the development of infection should be carefully examined, particularly in patients where the scar extends to the uterine cavity [6-8]. However, postoperative infections are typically early-onset, and the etiology of late-onset infections is not well understood.

One previous study has described how the vagina can act as a bacterial reservoir during the fecal-vaginal course of transmission in extraintestinal infections [9], which could hypothetically explain the late onset in the present case. However, we are uncertain when or how the infection developed, which precludes us from concluding that the pyogenic cyst was caused by late-onset infection secondary to the myomectomy. Another possibility is that the endometriosis was significantly associated with the infection, although it is surprising that no symptoms or inflammation markers were observed at the time of our initial diagnosis. This suggests that the infection did not occur immediately, and that the myomectomy scar was associated with the formation of endometriosis. Alternatively, a cervical endometriotic cyst might have progressed to pyogenic granuloma, or a gigantic cervical abscess might have developed *de novo* within the endometriosis. Patients should be carefully monitored for development of pyogenic cervical cysts; this case may indicate the currently unknown etiology of these cysts.

## Conclusions

We have reported the rare case of a pyogenic cervical cyst, containing signs of endometriosis and *E. coli* infection, in the vicinity of the uterine scar from previous open myomectomy. The previous surgery might be associated with the endometriosis at the site of infection, and endometriosis and/or infection might have contributed to the formation of the cyst.

### Consent

The patient gave written consent for the case report to be published.

## Abbreviations

CT: Computed tomography; MRI: Magnetic resonance imaging; SCC: Squamous cell carcinoma antigen

## Competing interests

The authors declare that they have no competing interests.

## Authors’ contributions

KO and YI treated the case and wrote the manuscript. DM and MF diagnosed the case pathologically. TA, KK, YO, and TF contributed to the diagnosis, obtained informed consent, and determined the management of the case. All authors read and approved the final manuscript.

## Pre-publication history

The pre-publication history for this paper can be accessed here:

http://www.biomedcentral.com/1472-6874/14/104/prepub
